# Nutritional Status as a Prognostic Factor for Survival in Palliative Care: A Retrospective Observational Analysis of Home Parenteral Nutrition in Cancer Patients with Inoperable Malignant Bowel Obstruction

**DOI:** 10.3390/nu16111569

**Published:** 2024-05-22

**Authors:** Karolina Tenderenda, Aleksandra Gierczak, Mariusz Panczyk, Jacek Sobocki, Zuzanna Zaczek

**Affiliations:** 1 Student Research Association for Clinical Nutrition, Department of General Surgery and Clinical Nutrition, Centre of Postgraduate Medical Education, Medical University of Warsaw, 00-401 Warsaw, Poland; 2 Department of Education and Research in Health Sciences, Faculty of Health Sciences, Medical Univeristy of Warsaw, 00-518 Warsaw, Poland; mariusz.panczyk@wum.edu.pl; 3 Department of General Surgery and Clinical Nutrition, Centre of Postgraduate Medical Education, 00-401 Warsaw, Polandzuzanna.zaczek@wum.edu.pl (Z.Z.); 4 Department of Human Nutrition, Faculty of Health Sciences, Medical University of Warsaw, Erazma Ciołka 27, 01-445 Warsaw, Poland

**Keywords:** palliative care patients, malignant inoperable bowel obstruction, home parenteral nutrition, malnutrition, survival time

## Abstract

Palliative care patients with malignant bowel obstruction are particularly at risk of developing malnutrition, which in turn directly shortens survival time and worsens quality of life (QoL). According to the available data, the survival time in this patient group is often less than three months. To avoid further complications related to malnutrition and poor outcomes in oncological therapy, nutritional therapy such as home parenteral nutrition (HPN) is offered. The aim of this study was to investigate whether nutritional status is a prognostic factor for survival in palliative care patients with malignant inoperable bowel obstruction qualified for home parenteral nutrition and which nutritional assessment tool has the most accurate prognostic value. This retrospective observational analysis included 200 patients with malignant bowel obstruction referred for home parenteral nutrition between January 2018 and August 2023. The analysis included laboratory test results, body mass index (BMI), Subjective Global Assessment (SGA), Nutritional Risk Index (NRI), Geriatric Nutritional Risk Index (GNRI), Prognostic Nutritional Index (PNI) and malnutrition as defined by the Global Leadership Initiative on Malnutrition (GLIM). The average survival time of the patients was 75 days. Patients with higher NRI and PNI scores were more likely to survive (NRI: *p* < 0.001; PNI: *p* < 0.001). The GLIM criteria, SGA scores and BMI values did not prove to be good prognostic factors for survival (GLIM *p* = 0.922, SGA *p* = 0.083, BMI *p* = 0.092). The results suggest that the use of NRI and PNI may be helpful in prognosing survival in these patients and that prevention of the development of malnutrition through earlier nutritional assessment and intervention should be considered in this patient group.

## 1. Introduction

The World Health Organization (WHO) defines palliative care as an approach that improves the quality of life of patients and their families facing problems associated with an untreatable illness [1]. If the treatment administered is ineffective and the patient’s recovery is prevented, palliative therapy should be considered. Palliative patients with disseminated abdominal and pelvic cancer are particularly susceptible to impairment of gastrointestinal function caused primarily by the disease but also by the treatment. Attention should also be paid to malignant bowel obstruction (MBO), which occurs in 6–13% of patients with gastric cancer [2] and 20–50% of patients with ovarian cancer, as well as in patients with colorectal [3] and pancreatic cancer, but may be a result of primarily extra-abdominal malignancy as well. Malignant bowel obstruction leads to complications such as chronic pain, bloating, nausea and vomiting, as well as the inability to eat [3]. This impairs the absorption of nutrients and leads to the deterioration of nutritional status and malnutrition. The consequences of malnutrition and not the disease itself are the cause of death in 10–20% of cancer patients [4]. MBO worsens the patient’s prognosis and makes palliative oncological treatment impossible. Moreover, water retention in palliative cancer patients disturbs several parameters, i.e., body mass and body mass index (BMI), and has a negative impact on survival [5]. In addition to chemotherapy, which is proven as a palliative therapy [6] to prolong survival [7], nutritional support should also be provided, giving a synergistic effect. In patients with malignant bowel obstruction, parenteral nutrition (PN) can be an effective method to improve the patient’s nutritional status, preferably administered as home parenteral nutrition. HPN improves prognosis and quality of life, and, in the majority of cases, makes oncological treatment reasonable [5]. Although HPN can be helpful in patients with malignant bowel obstruction, it should be borne in mind that patients qualified to receive this procedure require hospitalisation for days or weeks, during which the patient’s medical status is optimised, a central intravenous access is inserted, adequate training is conducted, and the composition of the nutritional admixture is adapted to the patient’s needs. The ESPEN guidelines [8] recommend that parenteral nutrition should be considered if survival is estimated to be greater than 2–3 months. However, this guideline is controversial due to the difficulty in estimating patient survival time. The question arises as to how accurately specialists can really determine the survival time of patients and whether there are tools that could facilitate this. In this study, attention was focused on nutritional status and nutritional risk assessment tools such as the Subjective Global Assessment (SGA) scale, laboratory tests, the Nutritional Risk Index (NRI), the Geriatric Nutritional Risk Index (GNRI) and the Prognostic Nutritional Index (PNI) to consider them as factors of survival for better assessment and decision making on possible nutritional treatment in palliative care patients with malignant bowel obstruction.

## 2. Materials and Methods

### 2.1. Setting and Study Design

This study was conducted at the Polish national reference centre for home parenteral nutrition (HPN) between January 2018 and August 2023. The research employed a retrospective observational review design, where data were collected from hospitalisation records during the HPN qualification and from all medical records of outpatient visits to the HPN centre. The study protocol received approval from the Ethics Committee of the Medical University of Warsaw (AKBE/348/2023), ensuring compliance with ethical standards in research.

### 2.2. Sample

**Figure 1:**
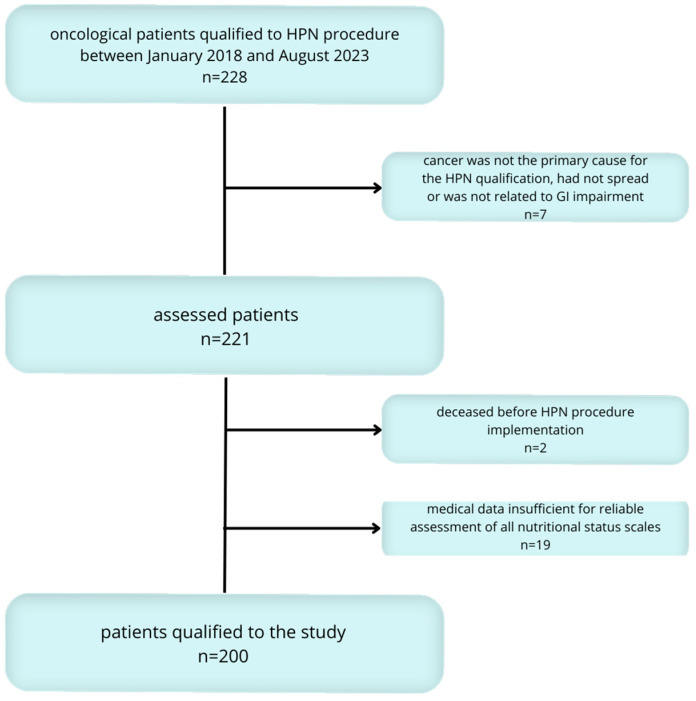
Flow chart: patients who met the inclusion criteria and qualified for the study group.

Participants included in this study were all consecutive adult palliative cancer patients with malignant bowel obstruction who were qualified for HPN treatment at the reference centre between January 2018 and August 2023. Eligibility for inclusion required that patients’ cancers were a direct cause of the qualification for home parenteral nutrition, with conditions not amenable to curative treatment due to advanced tumour progression and significant impairment of gastrointestinal function. Patients were excluded if their cancers had not spread, were not the primary cause of HPN qualification, or were not directly related to gastrointestinal function impairment. After applying these criteria, a total of 200 patients formed the study cohort. The detailed process of qualifying patients for the study is shown in Figure 1.

### 2.3. Approach to Home Parenteral Procedure

The criteria for eligibility for HPN included intestinal failure, the possibility of using parenteral nutrition (possibility of central venous access, care, patient co-operation), potential survival of more than 2 months, absence of treatment requiring hospitalisation (e.g., advanced pain management), and ethical reasons for further therapy (mainly quality of life). 

Qualification for home parenteral nutrition included a nutritional assessment, education and training of caregivers, a physical examination, blood tests, a prescription, and adjustment of a parenteral formula. If the patient did not already have a central intravenous access, a vascular port or tunnelled central venous catheter (CVC) was implemented. A parenteral nutrition formula was prepared individually by the hospital pharmacy or the patient’s caregiver according to the medical prescription. Ready-to-use (RTU) formulas were prescribed only to individual patients. 

The nutritional assessment included measurements of body weight and height, a full medical examination and screening of nutritional status based on the Subjective Global Assessment (SGA) and was performed by a trained clinical dietitian in the first 24 h after admission to the clinical nutrition unit. After discharge from hospital, patients had mandatory follow-up visits to the outpatient clinic every three months, or more frequently if adverse symptoms or complications arose. 

### 2.4. Data Collection and Nutritional Status Evaluation

Data collected included gender, age at the time of qualification for HPN, date of HPN commencement, date of death or withdrawal of HPN, type of cancer and stage of progression, anthropometric measurements (weight, height), BMI, unintentional weight loss in the 6 months prior to starting nutritional treatment, laboratory tests (serum albumin level, lymphocytes, leucocytes, total protein and C-reactive protein), data from the Subjective Global Assessment, information on the nutritional programme and the content of nutritional admixtures such as volume (mL), energy (kcal), osmolality, amino acids (g), fat (kcal), volume of fat emulsion (mL) and non-protein energy (kcal). 

BMI categories for patients < 65 years were classified according to the CDC criteria [9]: BMI < 18.5 kg/m^2^—underweight; BMI ≤ 25 kg/m^2^—normal weight; BMI ≥ 25 kg/m^2^—overweight. For patients over 65 years, Lipschitz criteria [6] were used: BMI < 22 kg/m^2^—underweight; BMI ≤ 27 kg/m^2^—normal weight; BMI ≥ 27 kg/m^2^—overweight. The results of the Subjective Global Assessment were interpreted as follows: A—well nourished; B—moderately (or suspected of being) malnourished; C—severely malnourished; D—high risk of malnutrition.

In addition to analysing the results of the SGA scale and BMI classification, the criteria of the Global Leadership Initiative on Malnutrition (GLIM) were used to assess the nutritional status of patients [10]. The loss of muscle mass was assessed using the physical examination data in the SGA scale and the decrease in the functional capacity of muscle mass (physical condition and abilities of the patients). The presence of inflammation was determined when the C-reactive protein (CRP) level exceeded 10 mg/L.

All of the following nutritional indexes were calculated during data collection and analysed by a second clinical dietitian. 

Based on serum albumin level, body weight and ideal body weight, the Nutritional Risk Index was calculated. Ideal body weight was calculated based on the Lorentz formula [11]:

Ideal body weight men = (height [cm] − 100 − ((height − 150)/4)

Ideal body weight women = (height [cm] − 100 − ((height − 150)/2)

The Nutritional Risk Index was calculated using the following formula [12]:

NRI = (1.519 × serum albumin) (g/L) + 41.7 × (present weight/ideal body weight)

The patients with an NRI score > 100 were considered to be at no nutritional risk, 97.5–100 as at mild risk, 83.5–97.5 at moderate and <83.5 at major nutritional risk. 

For patients who were over 65 years old, the Geriatric Nutritional Risk Index was calculated. The GNRI was calculated based on the formula proposed by Bouilanne et al. [13]:

GNRI = (1.489 × serum albumin) (g/L) + 41.7 × (present weight/ideal bodyweight)

The patients with a GNRI score > 98 were at no nutritional risk, ≤98 at low risk, 82 to <92 at moderate risk and <82 at major risk. 

Also, the Prognostic Nutritional Index was calculated based on the following formula [14]:

PNI = 10 × serum albumin (g/dL) + 0.005 × total peripheral lymphocyte count (mL)

The cut-off points for PNI were set as follows: PNI < 35—severe risk; PNI < 38—moderate risk; PNI > 38—normal risk. 

### 2.5. Statistical Analysis

Statistical analyses were conducted using STATISTICA version 13.3 (TIBCO Software, Palo Alto, CA, USA). Descriptive statistics summarised the demographic and clinical characteristics of the study population, with means and standard deviations (SDs) calculated for continuous variables like age, height, weight, and BMI, and the median (Mdn) and semi-interquartile range (IQR/2) reported where appropriate. Similar descriptive methods were applied for laboratory test results (including serum albumin, lymphocytes, leukocytes, CRP, and total protein) and for summarising data on patients’ nutritional programs (volume, energy, osmolarity, amino acids, fat content). The risk of malnutrition was assessed using indices such as SGA, NRS, PNI, Total Lymphocyte Count, NRI, and GNRI, presented in percentage format. Survival analysis utilised the Kaplan–Meier method, calculating survival time from the start of home parenteral nutrition. Multivariate Cox regression analysis explored predictors of patient survival, such as PNI and NRI/GNRI scores. Receiver Operating Characteristic (ROC) curves, utilising the Youden Index to establish the optimal cut-off points, determined these indices’ predictive accuracy for patient survival.

All tests were two-sided, and a *p*-value of less than 0.05 was considered statistically significant.

## 3. Results

A total of 200 patients met the inclusion criteria for this study. The mean age of the group was 58.9 ± 13 years (women 57.09, men 62.77; *p* = 0.004). The majority of patients included in the study were women—67.5%. All patients were classified according to tumour location as follows: 32.5% (*n* = 65) gynaecological cancers, 32.0% (*n* = 64) upper digestive tract cancers, 24% (*n* = 48) lower gastrointestinal tract cancers and 11.5% (*n* = 23) other cancers with a different or unknown origin. Detailed characteristic of the group, including age at qualification, height, weight, and body mass index, is presented in Table 1. 

In addition to the anthropometric examinations, the results of the laboratory tests were also taken into account, including serum albumin levels, lymphocytes, leucocytes, C-reactive protein and total protein, which are listed in Table 2. All laboratory tests were performed prior to the start of nutritional therapy at the patient’s admission. The C-reactive protein values showed that the majority of patients (93.0%) had severe inflammation on admission to hospital. The majority of them had a subclinical infection of the vascular port. 

All patients had individually compiled All-in-One (AIO) nutritional admixtures. One hundred and ninety-five patients (88.5%) received their nutritional admixture prepared and premixed in the hospital pharmacy. The data on the nutritional programme are shown in Table 3.

Assessment of nutritional status according to GLIM criteria showed that 88% (*n* = 176) of patients were malnourished on hospital admission. The results of the assessment of nutritional status and nutritional risk, assessed with SGA, PNI, Total Lymphocyte Count (TLC), NRI and GNRI, are shown in Table 4. 

Patients who were classified as “A” using the SGA (*n* = 1), “no risk” using the NRI (*n* = 4) and “normal” using the GNRI (*n* = 5) were not included in further analysis, and only patients who were at high risk of malnutrition or malnourished on admission to hospital were included.

**Figure 2:**
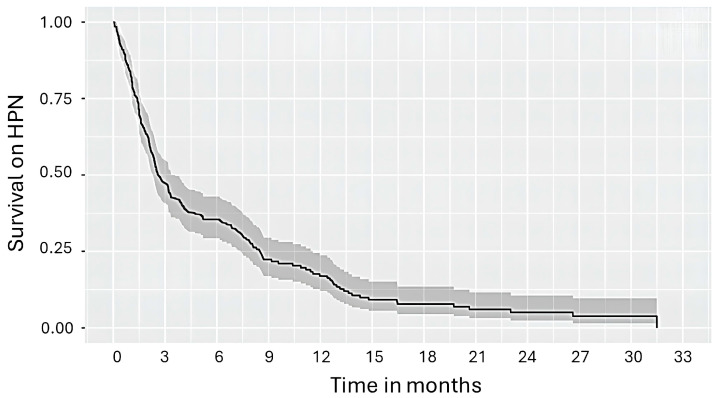
Kaplan–Meier curve for overall survival in palliative oncological patients with malignant bowel obstruction.

The overall survival (OS) curve for the study group is shown in Figure 2 and detailed data is presented in Table 5. 

The mean survival time of the patients was 75 days. The probability of survival was also assessed using PNI and NRI/GNRI scoring as possible predictors of patient survival. The results of multivariate Cox regression analysis showed that the Prognostic Nutritional Index score was positively associated with the risk of death (*p* < 0.001, HR = 0.950; 95%CI, 0.932–0.968).

**Figure 3:**
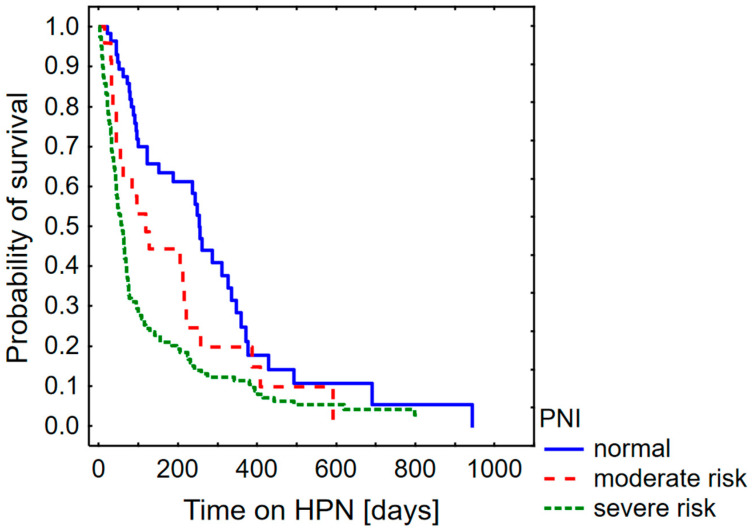
Probability of survival in palliative oncological patients with malignant bowel obstruction considering Prognostic Nutritional Index (PNI).

Figure 3 shows the probability of survival as a function of the Prognostic Nutritional Risk Index. The survival time in all figures is given in days.

As ESPEN states that the estimated survival time for patients included in the HPN procedure should be at least 90 days (three months), the relationship between the PNI score and the three-month survival time was investigated. The probability of three-month survival was 77.9% for patients classified with a “normal” PNI, 57.6% for “moderate risk” and 31.1% for “severe risk”.

**Figure 4:**
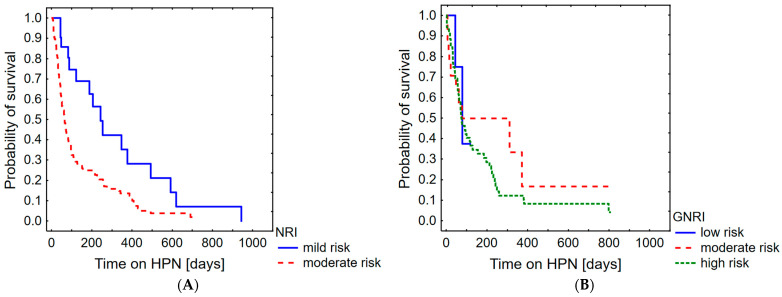
Probability of survival in palliative oncological patients with malignant bowel obstruction including NRI (**A**) and GNRI (**B**).

As the Nutritional Risk Index and Geriatric Nutritional Risk Index are age-dependent indicators of the risk of malnutrition, the analysed group was divided as follows: NRI, patients < 65 years old (*n* = 122); GNRI ≥ 65 years old (*n* = 78). NRI/GNRI scoring was also positively related to the risk of death (*p* < 0.001, HR = 0.979; 95%CI 0.969–0.990). Figure 4 shows the probability of survival as a function of the Nutritional Risk Index or Geriatric Nutritional Risk Index.

There was no significant correlation between the Geriatric Nutritional Risk Index values and the probability of survival; however, for the Nutritional Risk Index, the results showed that the “moderate” group had a significantly lower probability of survival than the “mild” group (*p* = 0.001). The probability of surviving three months was 74.6% in the mild-risk group and 38.8% in the moderate-risk group.

**Figure 5:**
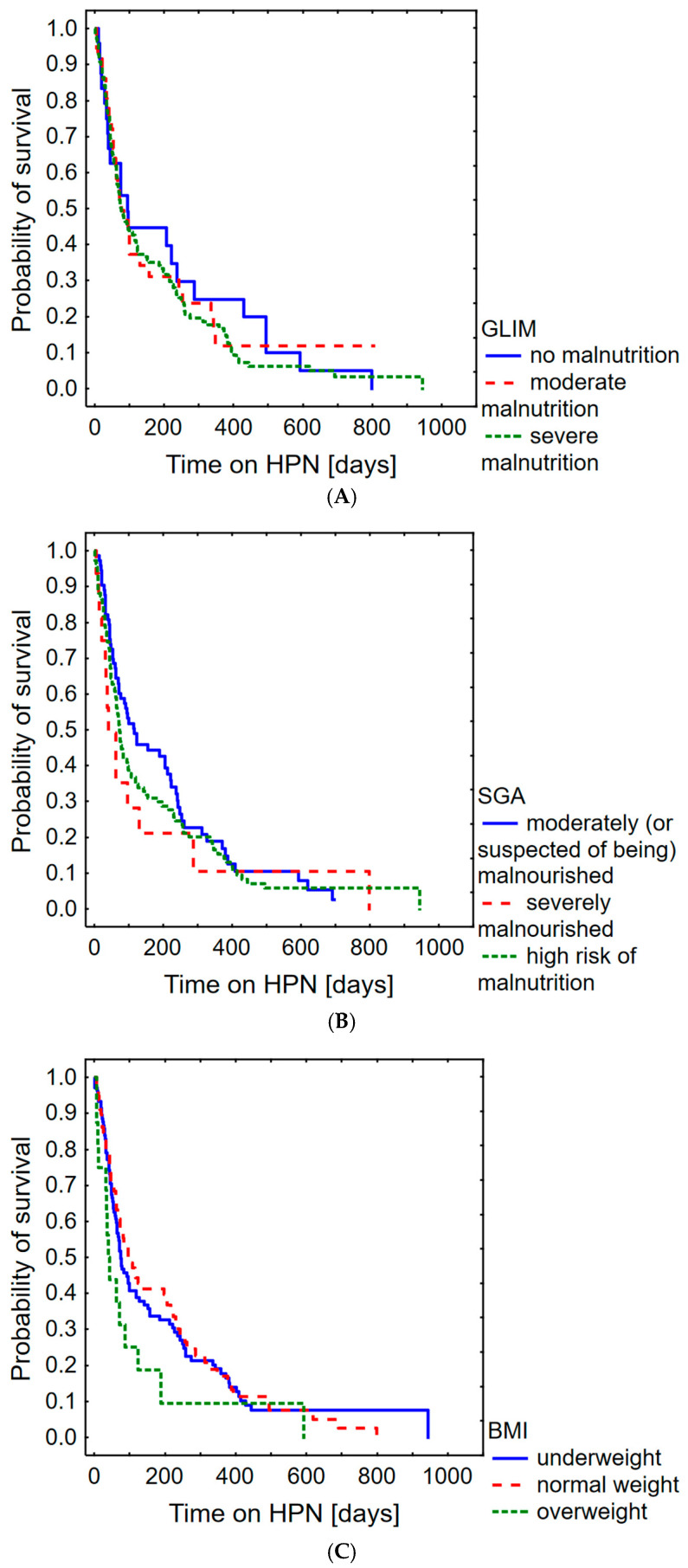
Probability of survival in palliative oncological patients with malignant bowel obstruction depending on GLIM (**A**), SGA (**B**) and BMI (**C**).

The presence of a relationship between nutritional status, taking into account criteria such as GLIM, SGA, BMI and survival time on home parenteral nutrition, was also analysed. The results for these criteria are shown in Figure 5. There was no significant difference between the groups; GLIM *p* = 0.922, SGA *p* = 0.083, BMI *p* = 0.092.

**Figure 6:**
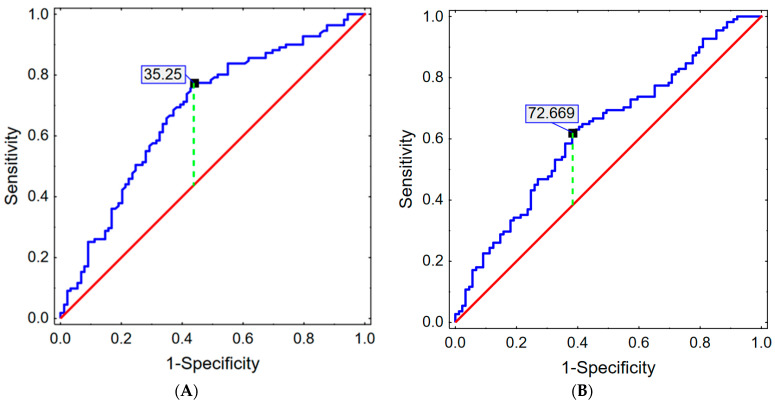
Receiver Operating Characteristic (ROC) curve for PNI (**A**) and NRI (**B**) scoring.

The ROC curves showed the best cut-off points for the Prognostic Nutritional Index and the Nutritional Risk Index, which are presented in Figure 6. For the PNI, the cut-off point was 35.25: *p* < 0.001 (AUC = 0.681; 95%CI 0.605–0.756). At this cut-off point, the test was shown to have a moderate ability to detect disease states (3-month survival) and a good ability to detect non-disease states (no 3-month survival). Further details on the ROC parameters can be found in Table 6.

For NRI, the cut-off point was 72.67: *p* = 0.001 (AUC = 0.626; 95%CI 0.549–0.704). Detailed data are presented in Table 7. 

## 4. Discussion

The main objective of the present study was to investigate whether the nutritional status of palliative cancer patients receiving home parenteral nutrition can be used as a prognostic factor for survival. According to a recent scoping review [15], clinical practice guidelines for nutritional assessment are not uniform with regard to the choice of specific tools and criteria. In this study, both the most commonly used tools, i.e., the body mass index and the Subjective Global Assessment, and those less commonly used in clinical practice, such as the GLIM criteria, the Nutritional Risk Index, the Geriatric Nutritional Risk Index and the Prognostic Nutritional Risk Index, were considered. The use of these indicators enabled not only an accurate assessment of the nutritional status of patients but also a review of whether they can be used as a tool to better estimate patient survival in the context of initiating nutritional therapy. 

In this study, 99.5% of patients were malnourished or at high risk of malnutrition according to the results of the SGA scale, and 88% of patients were malnourished based on the GLIM criteria. In the meta-analysis presented by Matsui et al. [10], which focused on treatment outcomes in cancer patients and was based on the GLIM criteria for malnutrition, the prevalence of malnutrition ranged between 30 and 40% in most studies. 

The ESPEN guideline (No. 7) for clinical nutrition in cancer stated that nutritional therapy should be implemented when patients are not yet severely malnourished [4]. This indicates an urgent need for more rapid nutritional interventions focusing on the assessment of nutritional status, particularly in the context of the impact of malnutrition on the treatment process. Another ESPEN recommendation (No. 4) states that parenteral nutrition should be implemented in patients with an estimated survival time of more than one to three months [16]. A meta-analysis by Naghibi et al. [17], which included patients with palliative malignancy and inoperable bowel obstruction (*n* = 244), found a mean survival time of 116 days. However, in our study, the median survival of patients with disseminated cancer in a palliative state was only 75 days, which does not meet the ESPEN criteria. 

It is also important to consider how we can improve the accuracy of survival time estimates. This study shows that neither BMI values nor Subjective Global Assessment scores are good tools for estimating survival time in the analysed patient group (BMI: *p* = 0.092, SGA: *p* = 0.083). Similar results for BMI were reported by Nazari et al. [18] and Przekop et al. [19] in patients with head and neck cancer. On the other hand, Chong et al. [20] reported that the modified Patient-Generated Subjective Global Assessment (mPG-SGA) is a valuable tool for survival prognosis in patients with malignant cancer involving two or more organs. This is consistent with the findings of Carvalho et al. [21], who reported that Patient-Generated SGA (PG-SGA) scores are directly related to survival in palliative cancer patients. The results of Huo et al. [22] show that the modified version has similar predictive power for survival in patients with lung cancer as the PG-SGA and the GLIM, suggesting that all three instruments are applicable in this patient group. This may suggest that the Patient-Generated Subjective Global Assessment (both basic and modified) is more suitable than the Subjective Global Assessment for prognosing survival prediction in the oncological population. Nevertheless, the study by Crestani et al. [23] showed that the SGA has good accuracy and sufficient specificity (>80%) in the diagnosis of malnutrition compared to the PG-SGA, demonstrating its usefulness in clinical practice.

The Global Leadership Initiative on Malnutrition criteria were also not found to be a good tool for predicting patient survival (*p* = 0.922). However, as shown in a meta-analysis by Yin et al. [24], GLIM-defined malnutrition has an impact on all-cause mortality in the oncological population, and these criteria are highly recommended for the assessment of nutritional status due to the use of both phenotypic and aetiological criteria [25], making them highly relevant in daily clinical practice. 

Although albumin levels are not a good indicator of nutritional status [26,27], they proved useful in this study as they were used to calculate indicators of nutritional status such as the Nutritional Risk Index, the Geriatric Nutritional Risk Index and the Prognostic Nutritional Index. This study showed significant results with regard to the NRI. Patients classified in the “moderate” group had a lower probability of survival than those classified as “mild risk” (*p* < 0.001). Similar conclusions were reached by Chaufour-Andre et al. [28] and Zhou et al. [29]. Both studies found that a low NRI was associated with poorer patient outcomes. A paper by Chen et al. [30], which focused on patients with breast cancer, highlighted that overall survival in this group was remarkably longer when the NRI was higher. It is therefore worth considering incorporating the Nutritional Risk Index into daily clinical practice, although it should be borne in mind that it only applies to patients under the age of 65. 

Particular attention should be paid to the Prognostic Nutritional Index, which is not used in daily practice. The analysis showed that patients with a “normal” PNI score had a 75.2% chance of surviving for three months. Zhang et al. [31] showed in their study of patients with gastrointestinal cancer that those with a higher PNI score had a higher survival rate (*p* < 0.001). Similar conclusions were drawn in Bullock et al.’s [32] paper, which focused on elderly patients (>70 years) with cancer. This suggests that the Prognostic Nutritional Index can be used to estimate patient survival with a lower margin of error. It should also be emphasised that the Prognostic Nutritional Index is a universal indicator compared to the Nutritional Risk Index, as it is applicable to any age group of adult patients. The use of NRI and PNI as indicators in the context of deciding whether to refer a patient for home parenteral nutrition should be considered, as this may minimise the risk of underdiagnosis and overtreatment.

It is generally recognised that the primary aim of nutritional therapy is to improve the patient’s nutritional status to such an extent that it also has a positive effect on the treatment outcome and QoL. However, it should not be forgotten that this is still a medical procedure that not only places a physical but also a psychological burden on the patient, requires hospitalisation to prepare for the procedure, and can be associated with complications that just as often end in hospitalisation and reduce the quality of life remaining [33]. It is also important to make an assessment after optimising the patient’s condition and to plan the optimal method of parenteral nutrition (especially AIO). The use of RTU admixtures and additional fluids leads to poorer outcomes as RTU is sub-optimally balanced and contains too much fat, and the separation of the nutritional mixture from the fluids shortens the infusion time and accelerates the water infusion time, leading to poorer bioretention and metabolism of macronutrients and having a tendency to cause oedema and water retention as a result of rapid fluid infusion.

Ultimately, the question arises as to where the ethical line is drawn between using nutritional therapy to improve the patient’s condition and another medical intervention that may shorten the patient’s final days. 

Our study had some limitations. Due to its retrospective design, we did not have access to detailed medical records from other medical institutions that included the date of diagnosis and the treatment given. In addition, some patients were transferred to hospice and were no longer in the care of the HPN centre (they were classified as weaned from HPN), so we had no current information on their survival.

## 5. Conclusions

This study has shown that there is a high need for early nutritional intervention, particularly in relation to the consequences of malnutrition, which directly increases the risk of poorer prognosis and death.

The nutritional status of the patient can be one of the most important elements in the assessment of estimated survival time and should be carried out with validated instruments.

The Nutritional Risk Index and Prognostic Nutritional Index have been shown to be good indicators for assessing the nutritional status of patients in relation to their survival time, although the PNI may prove to be the more versatile tool due to the lack of age restriction.

## Figures and Tables

**Table 1:** Characteristics of the study group including age, height, weight, and body mass index (BMI).

	N	M	SD	Mdn	IQR/2	Min	Max	CV [%]
Age at qualification	200	58.9	13.0	61.0	9.0	19.0	88.0	22.1
Height (cm)	200	166.9	9.1	165.0	7.0	145.0	190.0	5.4
Weight (kg)	200	56.6	11.7	55.0	8.5	30.4	86.0	20.7
BMI	200	20.3	3.9	19.8	2.4	12.7	32.8	19.4

M—mean; SD—standard deviation; Mdn—median; IQR/2—semi-interquartile range; CV—coefficient of variation.

**Table 2:** Characteristics of the study group including albumin level, lymphocytes, leukocytes, C-reactive protein (CRP) and total protein.

	N	M	SD	Mdn	IQR/2	Min	Max	CV [%]
Serum albumin (g/dL)	200	2.67	0.78	2.60	0.59	1.25	5.40	29.06
Lymphocytes (tys/μL)	200	1.28	0.66	1.17	0.39	0.16	4.56	51.71
% Lymphocytes	200	18.30	11.50	15.76	7.10	1.93	68.51	62.87
Leukocytes (tys/μL)	200	8.70	4.99	7.71	2.57	1.90	36.80	57.31
C-reactive protein (mg/L)	200	69.51	76.15	43.2	45.96	0.40	497.44	109.5
Total protein (g/dL)	198	6.41	1.04	6.40	0.72	3.10	8.90	16.20

M—mean; SD—standard deviation; Mdn—median; IQR/2—semi-interquartile range; CV—coefficient of variation.

**Table 3:** Characteristics of the study group including data related to the nutrition programme (volume, energy, osmolality, amino acids, calories of fat, volume of fat emulsion, non-protein energy).

	N	M	SD	Mdn	IQR/2	Min	Max	CV [%]
Volume (mL)	200	2452.8	377.7	2600.0	250.0	1000.0	3600.0	15.4
Energy (kcal)	200	1356.9	138.7	1360.0	59.3	715.0	1915.0	10.2
Mosm/L	200	844.0	131.2	824.0	58.9	145.0	1450.0	15.6
Amino acids (g)	200	49.1	7.7	50.0	7.3	17.0	62.5	15.6
Fat (kcal)	200	242.9	61.4	200.0	50.0	100.0	600.0	25.3
Volume of fat emulsion (mL)	200	129.9	35.6	100.0	25.0	50.0	300.0	29.0
Non-protein energy (kcal)	199	1155.6	145.8	1166.0	66.0	132.0	1665.0	12.6

M—mean; SD—standard deviation; Mdn—median; IQR/2—semi-interquartile range; CV—coefficient of variation.

**Table 4:** Summary of SGA, PNI, TLC, NRI and GNRI results.

Variable	N	%
SGA (*n* = 200)		
A	1	0.50
B	73	36.50
C	16	8.00
D	110	55.00
PNI (*n* = 200)		
normal	57	28.50
moderate risk	24	12.00
severe risk	119	59.50
Total lymphocyte Count (*n* = 200)		
nourished	29	14.50
moderate malnutrition	125	62.50
severe malnutrition	46	23.00
NRI (*n* = 122)		
no risk	4	3.28
mild risk	21	17.21
moderate risk	97	79.51
GNRI (*n* = 78)		
normal	5	6.41
low risk	4	5.13
moderate risk	17	21.79
high risk	52	66.67

**Table 5:** Details for Kaplan–Meier curve including 3–6–9–12-month survival.

				95% Confidence Interval	
Time	Number at Risk	Number of Events	Survival	Lower	Upper
3	89	104	47.3 %	40.8 %	54.9 %
6	61	22	35.5 %	29.3 %	42.9 %
9	34	21	22.3 %	16.9 %	29.5 %
12	24	8	16.9 %	12.1 %	23.7 %

**Table 6:** Parameters of ROC curve for Prognostic Nutritional Index (cut-off point = 35.25).

True Positives	False Positives	False Negatives	True Negatives	Sensitivity	Specificity	Accuracy	PPV	NPV	LR (+)	LR (−)	Youden Index
51	27	43	96	0.543	0.780	0.677	0.654	0.691	2.472	0.586	0.323

PPV—Positive Predictive Value; NPV—Negative Predictive Value; LR (+)—Positive Likelihood Ratio; LR (**−**)—Negative Likelihood Ratio.

**Table 7:** Parameters of ROC curve for Nutritional Risk Index (cut-off point = 72.67).

True Positives	False Positives	False Negatives	True Negatives	Sensitivity	Specificity	Accuracy	PPV	NPV	LR (+)	LR (−)	Youden Index
56	43	35	71	0.615	0.623	0.620	0.566	0.670	1.631	0.618	0.238

PPV—Positive Predictive Value; NPV—Negative Predictive Value; LR (+)—Positive Likelihood Ratio; LR (**−**)—Negative Likelihood Ratio.

## Data Availability

All statements and conclusions made within this article have been made with the data and information contained within the provided supplements (tables and figures). No other data were used.
